# Supplementary material for: Chromothripsis during telomere crisis is independent of NHEJ, and consistent with a replicative origin
Source: Genome Res. 2019 May;29(5):737–49. doi: 10.1101/gr.240705.118 (PMC6499312; doi:10.1101/gr.240705.118)
Supplement: Supplemental Material [file supp_gr.240705.118_Supplemental_file_1.zip › contigs/annotated_contigs/DB113/contig.2.DB113_length_746_mean_cov_11.654155496.docx]

**DB113_length_746_mean_cov_11.654155496**

```
CTGTCTCTCTCTTCTCCTACCCCTCCTTTCTCCCCTACTTTCTTCCTTTTGCTGTAGGTCCTTGAGAAAACTCTTGGTTCTTGGGTCTA
 >chr7:132960882-132961183 + E=3e-169
TGCCTTTAGTACCTAAAGGATAAAATGGGTTTGTAAGGAGTATTTTCATAATTGGCCAAAGGATGAACTGATCAAATAAGGGTTATGTA

GTATAACATTATAGTTATGTGGTTTTTAGCTGTATTTTTGAGATCACTCCTTTAATACCATTTAGTACTACTCTGTGATAAATAACAAT

AATTGCCACCACTTATTGAACACTGATTCTTT|GC|GCAGGGACCCTGAGCTGACCCCAGGCAAAGGTAGGATGGGCACATCCATCCAG
                                 >chr7:132826243-132826688 + E=7e-232
GCTGCTGACAGCCTCATCTGAGTCCCCCTGGAACTGATGTGCTCCAAACACCCCGATTCCCTGAAGCCAGACCTTGTGTCTTTGCTGCC

CTCTCCTCTATCAAAACTTGTTTTCAGACAGAATATCTGATGAACTTATTCAAACTGAAATGCTGACTTCCCTAGAATAATTATAACGA

ATTGTTCATTGCCTAGCTCCTTCCATTGGAATATAAGCTCCAGAAGGCTAGTGCTGCTTATGCCTTTACTTAACAGATTATCAATACAT

CTTTTTTGTTTTATTTTTATTTTTATATATTTATTTTGAGATCGAGTCTCCCCCTGTCCTCCAGCATGGAGTGCAGGGGCATGATCTTG

GCTGACTGCAACCTCCGCCCTCTGGGTTCCAGTG|CT
```
